# Supplementary material for: Characterization and Transcriptome Analysis of Maize Small-Kernel Mutant smk7a in Different Development Stages
Source: Plants (Basel). 2023 Jan 12;12(2):354. doi: 10.3390/plants12020354 (PMC9866416; doi:10.3390/plants12020354)
Supplement: Supplementary file 1 [file plants-12-00354-s001.zip › Supplementary Figures.docx]

**Supplementary Figures**


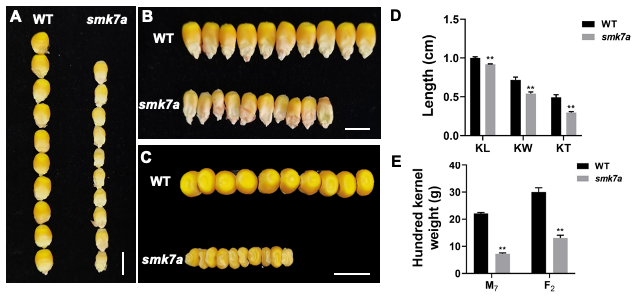


Figure S1 Comparison of the kernels traits of WT and *smk7a*. Phenotype comparison of the kernel length (A), width (B) and thickness (C) between WT and *smk7a*. Scale bar is 1cm. (D) Measurement of the kernel length (KL), width (KW) and thickness (KT) of WT and *smk7a*. (E) Comparison of the hundred kernel weight of the WT and *smk7a* from the seventh generation of the heterozygous mutant (*smk7a*/WT) and F_2_ generation. The values in (D) and (E) are the means of the three replicates ± SD. ** denotes statistical signiﬁcance with p < 0.01 by using a t-test when compared with the value in the WT.

Figure S2 GO enrichment analysis of the DEGs between WT and *smk7a* at 12 DAP (A) and 20 DAP (B). The red and purple bar represent the enrichment of the up-regulated and down-regulated DEGs, respectively. Y-axis shows the GO terms. X-axis shows the -log10 (p-value). The size of each dot represents the number of genes enriched in a GO term. Biological Process, BP; Cellular Component, CC; Molecular Function, MF.


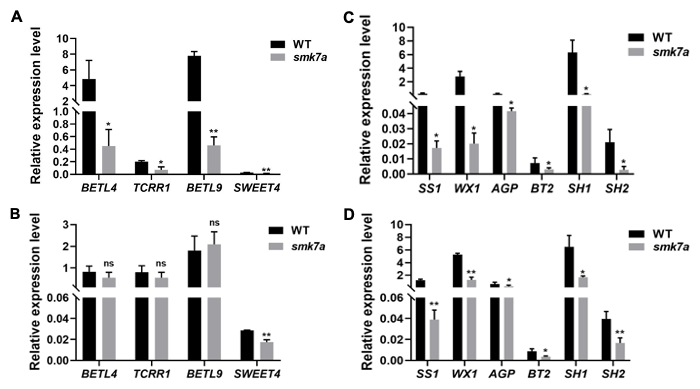


Figure S3 The qRT-PCR verification of the expression pattern of DEGs between WT and *smk7a*. Comparison of the expression level of the BETL-specific genes in WT and *smk7a* at 12 (A) and 20DAP (B). Comparison of the expression level of the starch and sucrose metabolism related genes in WT and *smk7a* at 12 (C) and 20DAP (D).
